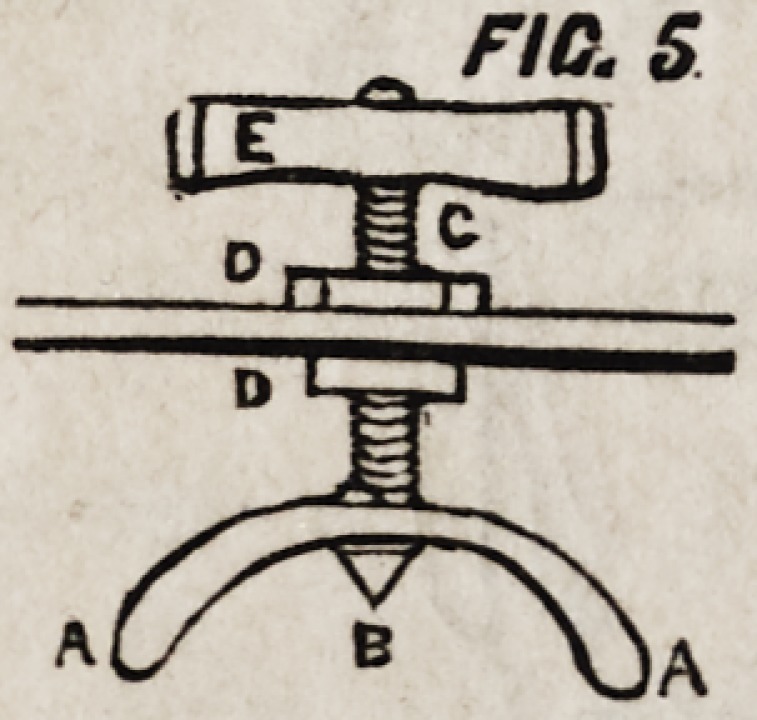# Alveolar Hemorrhage Compress

**Published:** 1852-10

**Authors:** R. Reid

**Affiliations:** Dentist, Edinburgh


### ARTICLE XVI.

*Alveolar Hemorrhage Compress, constructed by* DR. R. REID, Dentist, Edinburgh, with Engravings.

MEDICAL statistics record many cases of alveolar hemorrhage, resulting from the extraction of teeth, some of which have terminated fatally, notwithstanding every means having been resorted to that science could dictate or ingenuity suggest. A large number of cases, more or less difficult of control, are constantly occurring, that never find their way into medical periodicals; and most practitioners of ordinary standing will admit that in the course of their practice they have had alarming instances of this troublesome disease.

Hemorrhage, as the most formidable result of extraction, has engaged my attention for some time, with the view to devise a mode of meeting it successfully; and the result of my labors I now seek to lay before the medical profession and the public.

The nature and cause of this disease seem as yet to be enveloped in much obscurity, and no positive result has been arrived at as to whether the treatment ought to be constitutional or local. The agents usually employed are styptics, cold applications, cautery, and pressure. To discuss the relative merits of these, would be to travel beyond the limits of this paper, the last mentioned remedy—pressure—being its only object.

Pressure, when well regulated, uniform, and accurate, will be found at once the safest and most effectual remedy that can be employed. In every case that does not speedily yield to the application of styptics, it ought to be had recourse to before the discharge is becoming confirmed, and the soft parts surrounding the cavity deficient in vital energy. If it were necessary, a greater degree of pressure could then be employed without occasioning sloughing or ulceration. The directions laid down by Professor Miller in his able article on the treatment of Hemorrhagic Diathesis (Monthly Journal for July, 1842,) are at once so comprehensive and so simple, as to admit of being understood and acted upon by any one. The requisites to the accomplishing his mode are at hand in every house, and can be had without either difficulty or delay. In every ordinary case, this mode will answer the desired end as effectually as any apparatus that may be constructed, the only drawback being the necessarily immovable position of the lower jaw, which is to be "firmly shut and retained so immovably by turns of a bandage;" yet this could possibly be an objection in such cases only as might be deemed persistent.

The peculiar advantages of the alveolar hemorrhage compress which I have constructed, are as follows, namely:—

1st. Its easy adaptation to the size and shape of the head and face, and to the situation of the hemorrhage.

2d. Its attachment being on both sides of the face, no part of the instrument can be dislodged from the desired position.

3d. Sustenance can be administered to the patient without removal of the instrument, no obstacle being offered to the act of deglutition.

4th. Continuous and graduated pressure may be obtained, and the head may be reclined on either side without pain or inconvenience, while the motion of the lower jaw is very little impeded.

5th. Pressure can be applied on any number of bleeding points at once, on opposite sides of the jaw if required.

6th. In the apparatus for the under jaw, it has been so constructed as to press equally upon the inferior edge of the maxilla, from the symphysis to the angles, by which, and the use of a thick soft padding, all pressure upon the glands situated in the floor of the mouth are avoided. It also partakes of those advantages generally applicable to that for the upper jaw.

I have not sought to introduce this instrument to notice before satisfying myself of its practical utility. Its powers have been tested, and it has been found easy in application and effectual in operation, without proving irksome to the patient.

With these few imperfect remarks, I shall proceed to describe the application of the alveolar compress, as delineated on the engravings.

FIG. I.

FIG. 2.

Figures 1 and 2 represent the apparatus for the upper jaw. A is a sling composed of three leather straps passing across the frontal, epicranial, and occipital regions, and terminating in a ring at B, from which depends C, the attachment to the mouth-piece. These three straps are connected by one at D, passing in the mesial line over the vertex. E, the mouth-piece, of silver or plated metal, which is pulled into and retained in its position by the strap C.

Figures 3 and 4 show the under jaw. A the chin-plate formed of steel, and extending back nearly to the angles of the jaw, with a thick padding to prevent pressure on the glands situated in the floor of the

FIG. 3.

Fig. 4.

mouth; it is pierced with holes to admit the steel-rods B to pass down in a perpendicular direction, according to the size and arch of the inferior maxilla. B, the rods attaching the mouth-piece, which is retained in position by the screw-nuts C. D, the mouth-piece, with grooves E to admit the holder F traversing in the direction required.

Fig. 5

Figure 5 represents the holder with a portion of the mouth-piece. A the guard, which retains the pad of lint *in situ*, transfixed by the sharp point B. C the rod attaching it to the mouth-piece, the screws D keeping it firmly in its place. The nut E may be removed, and, if desired, the point covered with a piece of cork to protect the tongue.

In applying the compress, the socket is to be prepared in the usual way, by pressing a strip of lint into it, and putting a thick padding across it. When the cavity is not deep, I have been in the habit of inserting a piece of cotton saturated with a solution of mastic previous to applying the lint, the adhesive nature of the gum proving of service in retarding the flow of the blood.

I have seen Dr. Roberts' "apparatus for arresting hemorrhage after the extraction of teeth," and have no doubt it will be found useful. It appeared to me, however, regarding the apparatus for the upper jaw, that, as its attachment to the head was on one side only, "continued and steady" pressure at the desired point could not be obtained from it; and to retain the proper position but for a short time, required such a tightening of the horizontal strap as would prove insupportable. The movable nature of the scalp would also be a serious impediment to the retention of the instrument *in situ.*

In that for the under jaw the difficulty is increased, there being no contrivance to keep the pad from slipping off; and also, from its shape and limited dimensions, it will be apt to press upon the sublingual and maxillary glands.

The instrument is incapable of application on more than one bleeding point; and unless that it be in the front of the mouth, it will be impossible for the patient to lie on that side to which the instrument is applied, owing to the projection of the transverse and regulating bars in either instrument, more especially in that for the under jaw. In these views, however, I may be mistaken, and offer my opinion with diffidence.—*Monthly Jour. Med. Sci.*

## Figures and Tables

**FIG. 1. f1:**
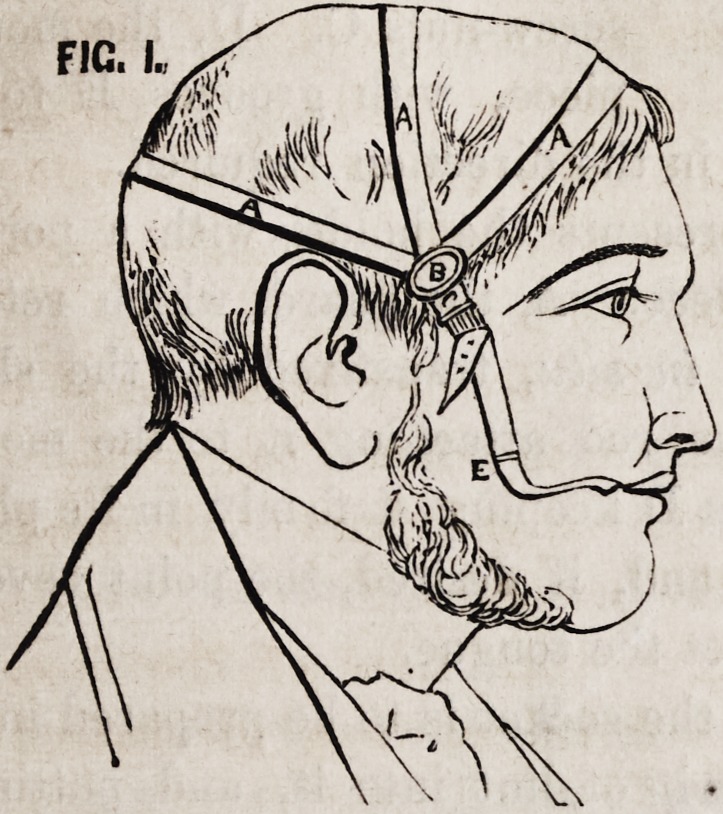


**FIG. 2. f2:**
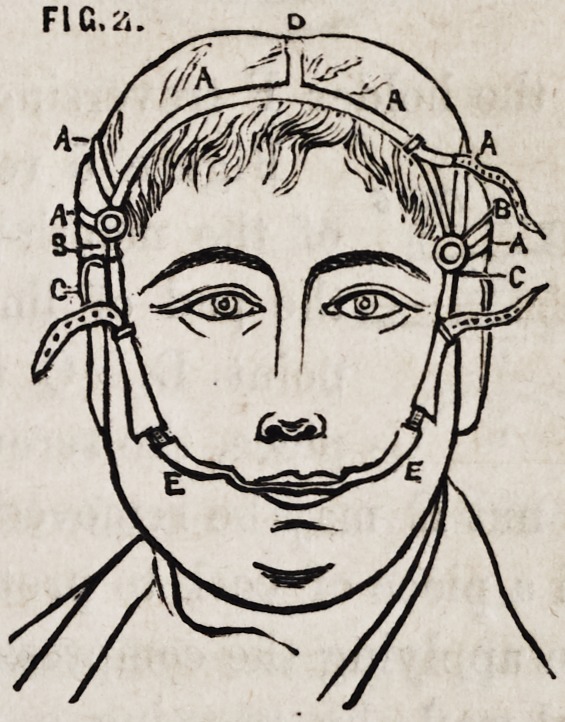


**FIG. 3. f3:**
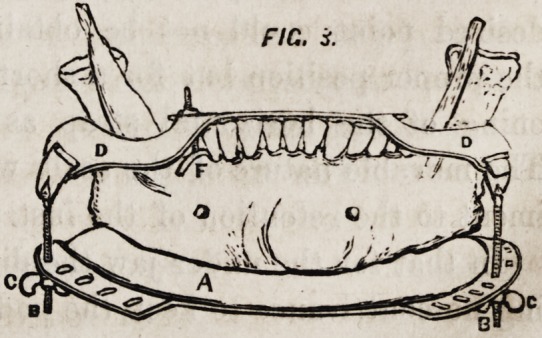


**Fig. 4. f4:**
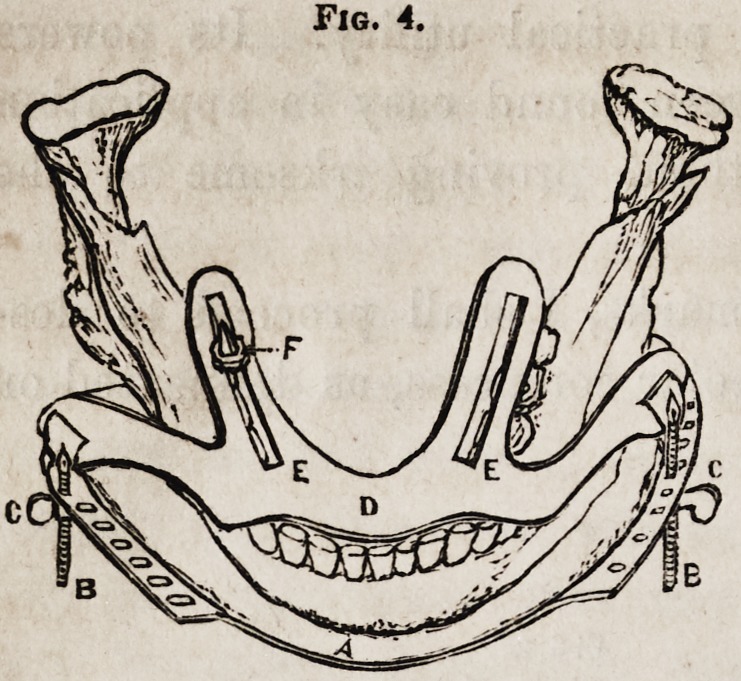


**FIG. 5. f5:**